# Use of Silence

**Published:** 1875-06

**Authors:** 


Use of Silence.—It is a pity that so few people understand the full effect of well-timed silence. How eloquent it is in reality! Acquiescence, contradiction, deference, disdain, embarrassment, and awe, may all be expressed by saying nothing. Should you hear an assertion which you may deem false, made by some one of whose veracity, politeness may withhold you from openly declaring your doubt, you denote a difference of opinion by remaining silent. Are you receiving a reprimand from a superior? You mark your respect by an attentive silence. Are you compelled to listen to the frivolus conversation of a fop? You signify your opinion of him by treating his loquacity by contemptuous silence. Again, how much domestic strife might have been prevented, how often might the quarrel which by mutual aggravation has perhaps, terminated in bloodshed, have been checked in the commencement by a judicious silence! Those persons only who have experienced them are aware of the beneficial effects of that forbearance, which, to the exasperating threat, the malicious sneer, or the unjustly imputed culpability, shall never answer a word. A soft answer turns away wrath; but sometimes erring humanity can not give this soft answer in moments of irritation: in such cases, there stands the fortress of silence, with doors wide open, as refuge for the tired spirit until calmer moments come. "Think of this seriously, you who glory in having "the last word."

